# Clinical complete response as a surrogate for pathological response in bladder cancer: a systematic review and meta‐analysis

**DOI:** 10.1111/bju.70214

**Published:** 2026-03-16

**Authors:** Navid Roessler, Marcin Miszczyk, Paolo Gontero, Alessandro Dematteis, Keiichiro Miyajima, Shota Inoue, Katharina Oberneder, Markus von Deimling, Victor M. Schuettfort, Malte W. Vetterlein, Bernadett Szabados, Pawel Rajwa, Pierre I. Karakiewicz, Jeremy Yuen‐Chun Teoh, Margit Fisch, Shahrokh F. Shariat

**Affiliations:** ^1^ Department of Urology Comprehensive Cancer Center, Medical University of Vienna Vienna Austria; ^2^ Department of Urology University Medical Center Hamburg‐Eppendorf Hamburg Germany; ^3^ Collegium Medicum, Faculty of Medicine, WSB University Dąbrowa Górnicza Poland; ^4^ Department of Urology Città della Salute e della Scienza, University of Torino School of Medicine Torino Italy; ^5^ Department of Urology Jikei University School of Medicine Tokyo Japan; ^6^ Department of Urology Okayama University Graduate School of Medicine, Dentistry and Pharmaceutical Sciences Okayama Japan; ^7^ Barts Experimental Cancer Medicine Centre, Barts Cancer Institute, Queen Mary University of London London UK; ^8^ Department of Urology University College London Hospital NHS Foundation Trust London UK; ^9^ Department of Urology Medical University of Silesia Zabrze Poland; ^10^ Cancer Prognostics and Health Outcomes Unit, Division of Urology University of Montréal Health Center Montréal, Québec Canada; ^11^ Department of Surgery S.H. Ho Urology Centre The Chinese University of Hong Kong Hong Kong China; ^12^ Department of Urology Weill Cornell Medical College New York NY USA; ^13^ Department of Urology University of Texas Southwestern Dallas TX USA; ^14^ Department of Urology Second Faculty of Medicine, Charles University Prague Czech Republic; ^15^ Hourani Center for Applied Scientific Research, Al‐Ahliyya Amman University Amman Jordan; ^16^ Karl Landsteiner Institute of Urology and Andrology Vienna Austria

**Keywords:** muscle‐invasive bladder cancer, clinical complete response, pathological complete response, bladder‐sparing strategies, neoadjuvant systemic therapy

## Abstract

**Objective:**

To evaluate the concordance between clinical complete response (cCR) and pathological complete response (pCR) in muscle‐invasive bladder cancer (MIBC) to assess the surrogacy and prognostic value of cCR for guiding bladder‐sparing strategies.

**Methods:**

In this prospectively registered systematic review and meta‐analysis (CRD420251066540), we searched MEDLINE, EMBASE, and Web of Science in June 2025 for studies reporting clinical and pathological complete response rates in patients with MIBC undergoing neoadjuvant therapy followed by radical cystectomy (RC). Pooled concordance was estimated via random‐effects meta‐analysis. Risk‐of‐bias was assessed using the Risk Of Bias In Non‐randomised Studies of Interventions (ROBINS‐I).

**Results:**

Out of 1947 individual records, 10 (*n* = 894) retrospective and three (*n* = 181) prospective studies comprising 1075 patients were included. Restaging modalities for cCR assessment included transurethral resection of the bladder (TURB; *n* = 188, two studies), computed tomography (*n* = 221, two studies), magnetic resonance imaging (MRI; *n* = 122, two studies), and fluorodeoxyglucose positron emission tomography (*n* = 45). One study (*n* = 56) used perioperative cystoscopy, while the remaining five studies (*n* = 499) combined imaging with cystoscopy or TURB. The concordance (*n* = 779, nine studies) between cCR and pCR was 0.51 (95% confidence interval [CI] 0.42–0.60), the concordance (*n* = 536, seven studies) between non‐cCR and non‐pCR was 0.84 (95% CI 0.70–0.92). Most studies were rated as having moderate concerns regarding bias, and one as serious due to examiner‐dependent bias of cystoscopy‐based cCR assessment.

**Conclusion:**

Current evidence does not support relying on the current definition of cCR alone, which poorly predicts pCR, to guide treatment decisions. Ongoing trials assessing the combination of MRI plus TURB with urine and/or blood based circulating tumour DNA may help refine cCR evaluation and support the sole introduction of bladder‐sparing approaches in patients with MIBC who respond to neoadjuvant systemic therapy.

**Abbreviations**
cCR: clinical complete response
ctDNA: circulating tumour DNA
DFS: disease‐free survival
FDG PET: fluorodeoxyglucose positron emission tomography
MFS: metastasis‐free survival
MIBC: muscle‐invasive bladder cancer
mpMRI: multiparametric MRI
MVAC: methotrexate, vinblastine, doxorubicin (adriamycin) and cisplatin
NAST: neoadjuvant systemic therapy
NAC: neoadjuvant chemotherapy
OR: odds ratio
OS: overall survival
PC: partial cystectomy
pCR: pathological complete response
RC: radical cystectomy
ROBINS‐I: Risk Of Bias in Non‐randomised Studies of Interventions
TMT: trimodal therapy
TURB: transurethral resection of bladder
utDNA: urinary tumour DNA
ypT: pathological tumour stage after neoadjuvant therapy

## Introduction

The current standard for patients with non‐metastatic muscle‐invasive bladder cancer (MIBC) is cisplatin‐based neoadjuvant chemotherapy ± neoadjuvant systemic therapy (NAST) followed by radical cystectomy (RC) [1, 2]. NAST prior to RC improves survival outcomes compared to RC alone [3, 4, 5], without significant increase in complications and adverse events [6, 7].

Pathological complete response (pCR), defined as the absence of residual tumour (pathological tumour stage after neoadjuvant therapy [yp]T0) in the RC specimen following NAST, is an established surrogate marker of improved survival [8, 9]. However, reliance on post‐RC pathology for pCR assessment precludes its use in clinical decision‐making [10]. In contrast, clinical complete response (cCR) represents a potentially clinically relevant intermediate endpoint that, if accurately defined and validated, could support bladder‐sparing (BSP) strategies in well selected patients [11, 12]. Multimodal assessment of cCR has been associated with favourable oncological outcomes and may provide a basis for safely implementing BSP approaches [13].

However, current restaging approaches after NAST remain highly variable, lacking standardisation and validation thereby limiting the clinical utility of cCR [14]. BSP strategies promise some oncological efficacy while delivering improved functionality and health‐related quality of life compared to RC by avoiding overtreatment. To ensure safe selection of patients for BSP, a valid, reliable, easily obtainable, and accurate successful endpoint post‐NAST is necessary.

In this systematic review and meta‐analysis, we aimed to assess the concordance between cCR and pCR following NAST prior to RC in patients with MIBC.

## Methods

This systematic review and meta‐analysis was registered with the International Prospective Register of Systematic Reviews (CRD420251066540) and conducted in accordance with the Preferred Reporting Items for Systematic Reviews and Meta‐analyses (PRISMA) flowchart (File 1 in Data S1), the A MeaSurement Tool to Assess systematic Reviews 2 (AMSTAR 2) checklist (File 8 in Data S1), and recent guidelines for systematic reviews and meta‐analyses [15, 16, 17].

### Study Selection

The research question and inclusion criteria were defined using the population, intervention, comparison, outcome, and study design (PICOS [Population, Intervention, Comparison, Outcome, and Study Design]) framework (File 7 in Data S1). We searched MEDLINE (via PubMed), EMBASE and Web of Science Core Collection for studies evaluating patients with MIBC who underwent clinical restaging to assess cCR after at least one cycle of systemic neoadjuvant therapy prior to RC. We included all retrospective and prospective reports, including subset and *post hoc* analyses, that reported pCR rates among patients with cCR. To maintain cohort homogeneity, studies restricted to performing partial cystectomy (PC) were excluded. Non‐English language manuscripts, studies not providing original data, editorials, and review articles were also excluded.

The search strategy was performed in June 2025, and the detailed search strategy is provided in File 2 in Data S1. Reports were merged and de‐duplicated using EndNoteX9 (Clarivate) and the title‐abstract screening was conducted independently by two authors. Following title‐abstract screening, full‐text reports were retrieved and screened for relevance independently. Backward citation searching was performed to identify potentially relevant additional records. At each step of the review, conflicts were resolved through consensus among co‐authors.

### Data Extraction

Two authors independently extracted data, including the first author's name, year of publication, patient characteristics (e.g., age, sample size, neoadjuvant therapy regimen), restaging details (e.g., type of restaging modality [cystoscopy, urine cytology, transurethral resection of the bladder (TURB), CT, MRI, fluorodeoxyglucose positron emission tomography/CT (FDG PET/CT)]), and rates of cCR and pCR (defined as cT0N0 and ypT0N0). All conflicts that arose during the data extraction process were resolved through discussion between authors.

### Risk of Bias Assessment

Each study was evaluated independently by two authors using the Risk Of Bias in Non‐randomised Studies of Interventions (ROBINS‐I) tool for non‐randomised studies [18].

### Statistical Analysis

A random‐effects meta‐analysis using logit‐transformed proportions was conducted to estimate the pooled pCR rate among cCR patients after neoadjuvant therapy prior to RC. Between‐study heterogeneity was assessed using the *I* ^2^ statistic (with >50% indicating substantial heterogeneity), and *τ* ^2^ was estimated using the restricted maximum likelihood (REML) method. Sensitivity analyses, including leave‐one‐out procedures, were performed to evaluate the influence of individual studies. Results were visualised using forest and funnel plots. All analyses were two‐sided and conducted in R (R Foundation for Statistical Computing, Vienna, Austria).

## Results

Based on our inclusion criteria, 13 studies comprising 1075 patients were included for this systematic review and meta‐analysis [19, 20, 21, 22, 23, 24, 25, 26, 27, 28, 29, 30, 31]. Among these, three studies (*n* = 181) were prospective in design [20, 23, 25], and 10 studies (*n* = 894) were retrospective. The clinical stage at diagnosis prior to neoadjuvant therapy was predominantly T2. The most commonly administered neoadjuvant regimen was gemcitabine/cisplatin, with the majority of patients receiving three to four treatment cycles. Two studies (*n* = 188) assessed TURB for restaging prior to RC [21, 23]. Five studies (*n* = 388) used imaging modalities including CT (*n* = 221, two studies), MRI (*n* = 122, two studies), or FDG PET (*n* = 45, one study) for post‐NAST restaging evaluation [19, 22, 24, 27, 28]. One study (*n* = 56) conducted perioperative cystoscopy before surgery [20]. Additionally, five studies (*n* = 499) combined imaging techniques (CT or MRI) with cystoscopy or TURB for restaging [25, 26, 29, 30, 31]. Table 1 summarises the characteristics of the included studies [19, 20, 21, 22, 23, 24, 25, 26, 27, 28, 29, 30, 31].

**Table 1:** Demographics and clinical characteristics of the included studies.

References	Patients, *n*	Age, years	Clinical Stage at diagnosis, *n* (%)	Neoadjuvant therapy regime, *n* (%)	Number of neoadjuvant therapy cycles, *n* (%)	Restaging modality	cCR, *n/N*	pCR, *n/N*	True‐positive (cCR = pCR)
Asad et al., 2022 [20]	56	Median 70 (range 39–80)	T2: 43 (77); T3: 12 (21); T4a: 1 (2)	HD‐MVAC: 48 (86); MVAC: 2 (4); Gem/Cis: 5 (9); Gem/Car: 1 (2)	Two: 6 (11); three: 45 (80); four: 4 (7); six: 1 (2)	Cystoscopy	28/56	17/56	14/28
Becker et al., 2021 [21]	114	Median 68 (IQR 62–71)	NR	Gem/Cis: 74 (65); MVAC: 4 (4); dd‐MVAC: 10 (9); Gem/Car: 6 (5); other 20 (18)	≤Two: 7 (6); ≥three: 90 (79); NR: 17 (15)	TURB	53/114	28/114	25/53
deVere White et al., 2009 [23]	74	Median 69 (range 49–83)	T2: 52 (70); T3: 17 (23); T4a: 5 (7)	Gem/Car/Pacli: 74 (100)	Three: 74 (100)	TURB	34/74	4/35	4/10
Alam et al., 2023 [19]	141	Median 65	NR	MVAC: 37 (26.3); Gem/Cis: 79 (56); other: 25 (17.7)	NR	CT	58/141	39/141	17/58
Mogos et al., 2020 [27]	80	Median 68 (range 44–80)	T2: 31 (39); T3: 38 (47); T4a: 11 (14)	MVAC and HD‐MVAC: 75 (94); Gem/Cis: 5 (6)	One: 1 (1); two: 9 (11); three: 46 (58); four: 23 (29); five: 1 (1)	CT	36/71	31/71	14/36
De Maeseneer et al., 2024 [22]	40	NR	NR	ddMVAC: 40 (100)	One: 1 (2.5); two: 2 (5); three: 2 (5); four: 35 (88)	mpMRI	6/40	11/40	4/6
Necchi et al., 2020 [28]	82	NR	NR	Pembrolizumab: 82 (100)	Three: 82 (100)	mpMRI	37/79	30/82	23/37
Fitoussi et al., 2023 [24]	45	Mean 66 (range 50–74)	NR	AMVAC: 26 (58); GemCis/Car: 19 (42)	Six: 26 (58) [AMVAC]; Three: 19 (42) [GemCis/Car]	FDG PET/CT	34/45	21/45	20/34
Kim et al., 2023 [25]	51	Median 66 (range 48–84)	T2: 33 (65); T3: 13 (26); T4a: 5 (9.8)	Gem/Cis/Nivolumab: 51 (100)	Three to four: 51 (100)	CT or urine cytology and cystoscopy	30/51	12/34	13/18
Meyer et al., 2014 [26]	109	Mean 68.3 (SD 9.6) [*n* = 32, cT0]	T2: 30 (29); T3: 2 (2)	MVAC: 21 (19); Gem/Cis: 8 (7.3); Gem/Car: 2 (2); Car: 1 (1)	NR	CT, urine cytology, and TURB	32/109	5/14	4/7
Piao et al., 2024 [29]	161	Mean 64.5 (SD 8.7)	T2: 138 (86); T3: 19 (12); T4: 4 (2.5)	Gem/Cis: 161 (100)	Three: 72 (45); four: 89 (55)	TURB, urine cytology, and CT	161/161	103/161	103/161
Reese et al., 2014 [30]	62	Mean 64.4	T1: 3 (5); T2: 36 (58); T3: 21 (34); T4: 2 (3)	Gem/Cis: 47 (76); Gem/Car: 3 (5); Cis/Eto: 2 (3); MVAC: 2 (3); Pacli/Car: 2 (3); Pacli/Gem: 1 (2); other: 5 (8)	≥Three: 61 (100)	Chest X‐ray or CT, bimanual examination, and cystoscopy (optional with TURB)	22/62	12/48	8/21
Scattoni et al., 1995 [31]	60	Mean 56.1 (38–75)	T2: 2 (0.3); T3a: 20 (33); T3b: 29 (48); T4: 9 (1.5)	CMV: 60 (100)	≥Two: 60 (100)	CT or MRI, cystoscopy, urine cytology, and ultrasound	6/60	5/60	3/6

AMVAC, accelerated MVAC; CMV, cisplatin, methotrexate, vinblastine; ddMVAC, dose‐dense MVAC; Gem/Car, gemcitabine/carboplatin; Gem/Cis, gemcitabine/cisplatin; HD‐MVAC, high‐dose MVAC; IQR, interquartile range; NR, not reported; Pacli, paclitaxel.

Percentages may not add up to 100%, as they are rounded.

### Assessment of Risk of Bias

The risk of bias judgements of each domain for each included study are summarised in File 6 in Data S1. According to ROBINS‐I tool for non‐randomised studies, three studies were rated as having a low risk of bias [25, 29, 31], and nine studies were assessed as moderate risk [19, 21, 22, 23, 24, 26, 27, 28, 30], primarily due to the assessment of cCR using only a single restaging modality and the retrospective study design. One study was rated as having a serious risk of bias because the cCR was assessed immediately before surgery solely by cystoscopy [20], which may introduce substantial bias due to its examiner dependency.

### The cCR and pCR in the Included Studies

Overall, eight studies (*n* = 632) assessed a single restaging modality (cystoscopy, TURB, CT, MRI, or FDG PET) [19, 20, 21, 22, 23, 24, 27, 28], whereas the remaining five studies (*n* = 443) evaluated a combination of imaging techniques (CT or MRI) with cystoscopy or TURB for restaging [25, 26, 29, 30, 31].

#### Single Restaging Modality: cCR and pCR

Asad et al. [20] (2022) prospectively evaluated the diagnostic accuracy of visual cCR assessment via cystoscopy after NAST, predominantly with dose‐dense high‐dose methotrexate, vinblastine, doxorubicin (adriamycin) and cisplatin (MVAC), in patients undergoing RC (*n* = 56). Cystoscopy was performed in the operating room immediately prior to RC. The cCR was visually suspected in 50% (28/56) of patients, while pCR was achieved in 30% (17/56). Among patients with cCR, 50% (14/28) also achieved pCR. Becker et al. [21] retrospectively analysed the diagnostic accuracy of TURB after predominantly cisplatin‐based neoadjuvant chemotherapy (NAC) regimens, including gemcitabine/cisplatin, standard‐dose MVAC, and dose‐dense MVAC, in patients undergoing RC (*n* = 114). A cCR was observed in 47% (53/114) of patients, while pCR was achieved in 25% (28/114). Among patients with cCR, 47% (25/53) also achieved pCR, corresponding to a false‐positive rate of 53%. DeVere White et al. [23] (2009) conducted a phase II trial evaluating a sequential NAC regimen of paclitaxel, carboplatin, and gemcitabine followed by restaging TURB and optional RC (*n* = 74). A cCR was observed in 46% (34/74) of patients. Of those with cCR, 29% (10/34) proceeded to immediate RC, with pCR confirmed in 40% (4/10), indicating a false‐positive rate of 60% (6/10). Survival analysis showed a 2‐year overall survival (OS) estimate of 59%, with improved survival in patients achieving cCR (75%). Alam et al. [19] retrospectively assessed the predictive value of CT in patients with MIBC following NAC with MVAC or gemcitabine/cisplatin prior RC (*n* = 141). Post‐NAC CT was used to evaluate cCR and was observed in 41% (58/141) of patients, while pCR was achieved in 28% (39/141). Among patients with cCR, 29% (17/58) had a corresponding pCR, indicating a false‐positive rate of 71%. No significant correlation between cCR and pCR was found (odds ratio [OR] 1.13; *P* = 0.7; Cohen's *κ* = 0.03), reflecting minimal agreement. Post‐NAC CT showed low sensitivity (44%) and specificity (60%) for predicting pCR. Mogos et al. [27] retrospectively assessed the prognostic value of pre‐final‐cycle CT imaging in patients with MIBC undergoing NAC (mostly MVAC or dose‐dense MVAC) followed by RC (*n* = 80). A cCR was observed in 51% (36/71) of patients, while pCR was achieved in 44% (31/71). Among those with cCR, 39% (14/36) had a corresponding pCR, indicating a false‐positive rate of 61%. CT imaging before the final NAC cycle demonstrated limited diagnostic performance, with a sensitivity of 64% and specificity of 36% for the detection of non‐responders. A cCR did not significantly correlate with 2‐ or 3‐year OS (2‐year OS: OR 0.64, 95% CI 0.16–2.58; 3‐year OS: OR 0.73, 95% CI 0.21–2.57). In contrast, pathological response was significantly prognostic: patients with stable or progressive pathological disease had worse 2‐year OS compared to those with pCR (OR for death in patients with stable disease 7.66, 95% CI 2.03–28.98; and in those with progressive disease 8.96, 95% CI 1.93–41.56). De Maeseneer et al. [22] (2024) retrospectively analysed the diagnostic accuracy of multiparametric MRI (mpMRI) in predicting pCR after NAC with dose‐dense MVAC in patients undergoing RC (*n* = 40). A cCR was observed in 15% (6/40), while pCR was achieved in 28% (11/40) of the patients. Among those with cCR, 67% (4/6) patients were pCR, corresponding to a false‐positive rate of 33%. The overall accuracy of mpMRI to predict pCR was 78%, with specificities of 93% (NAC Vesical Imaging‐Reporting and Data System [VI‐RADS]) and 96% (three‐step score), but with low sensitivities of 36% for both methods. Necchi et al. [28] (2020) evaluated mpMRI as a predictive tool for pCR after neoadjuvant pembrolizumab in patients with MIBC undergoing RC (*n* = 82). Among 79 patients with available data, 47% (37/79) showed cCR. Of these, 62% (23/37) achieved corresponding pCR, indicating a false‐positive rate of 38%. Univariable logistic regression demonstrated that mpMRI‐defined cCR was strongly predictive of pCR (OR 9.6, 95% CI 3.4–31; *P* < 0.001) in internal and (OR 9.8, 95% CI 3.4–31; *P* < 0.001) external validation; however, the area under the curve for predicting pCR was moderate, with 0.76 (95% CI 0.68–0.83) and 0.74 (95% CI 0.66–0.82) for internal and external validation, respectively. Fitoussi et al. [24] (2023) evaluated the accuracy of FDG PET/CT for response assessment in patients with MIBC following NAC with dose‐dense accelerated MVAC or gemcitabine/cisplatin prior RC (*n* = 45). A cCR was observed in 76% (34/45) of patients. A pCR was confirmed in 47% (21/45) of patients following RC. Among patients with cCR, 59% (20/34) had a corresponding pCR, resulting in a false‐positive rate of 41%. FDG PET/CT demonstrated high sensitivity of 95% and negative predictive value of 91% but had poor specificity and positive predictive value (42% and 59%, respectively).

#### Combination of Restaging Modalities: cCR and pCR

Kim et al. [25] (2023) prospectively investigated the efficacy of neoadjuvant nivolumab plus gemcitabine/cisplatin in patients with MIBC eligible for RC (*n* = 51). cCR was assessed using CT imaging or urine cytology combined with cystoscopy and was observed in 59% (30/51) of patients. pCR was confirmed in 24% (12/51) of all patients and in 35% (12/34) of those who underwent RC. Among patients with cCR who proceeded to surgery, 72% (13/18) had a corresponding pCR, resulting in a false‐positive rate of 28%. The median follow‐up was 24 months, with disease‐free survival (DFS) rates of 90% at 12 months and 73% at 24 months. Patients achieving cCR had significantly improved DFS (hazard ratio 0.16, 95% CI 0.04–0.57). Meyer et al. [26] (2014) retrospectively evaluated the outcomes in patients with cCR following platinum‐based NAC, including MVAC and gemcitabine/cisplatin, eligible for RC (*n* = 109). cCR was defined by negative post‐NAC cystoscopy (with deep TURB biopsies), urine cytology, and cross‐sectional imaging (CT). Post‐NAC, 29% (32/109) of the patients achieved cCR. The cCR rate varied by NAC regimen, with 37% for MVAC, 29% for gemcitabine/cisplatin, and 20% for gemcitabine/carboplatin (*P* = 0.063). In total, 14 cCR patients underwent RC (immediate or delayed), with an overall pCR rate of 36% (5/14). The true‐positive rate among immediate RC patients was 57% (4/7), indicating a false‐positive rate of 43%. Piao et al. [29] (2024) performed a retrospective study of patients with MIBC who achieved cCR after NAC with gemcitabine/cisplatin and subsequently underwent RC (*n* = 161). cCR was assessed by repeat endoscopic examination with biopsies of previously involved and normal‐appearing mucosa, urine cytology, and cross‐sectional imaging (CT). Among these patients, 64% (103/161) achieved pCR after RC, yielding a true‐positive rate of 64% (103/161) and a false‐positive rate of 36%. Reese et al. [30] (2014) retrospectively analysed patients with MIBC who received NAC with gemcitabine/cisplatin or MVAC followed by RC (*n* = 62). Post‐NAC restaging included cross‐sectional imaging (CT), endoscopic bladder evaluation, bimanual examination, and biopsy of visible lesions when indicated. cCR was observed in 36% (22/62) of patients, whereas pCR was achieved in 25% (12/48). Among the 21 patients with cCR who proceeded to RC, 38% (8/21) had pCR. Agreement between post‐NAC clinical stage and pathological stage was low but stronger (Cohen's *κ* = 0.17) compared to initial clinical staging (*κ* = 0.02). Post‐NAC clinical stage was moderately but significantly associated with advanced pathological stage, whereas age, sex, smoking history, pre‐NAC clinical stage, and NAC regimen were not. Scattoni et al. [31] (1995) retrospectively evaluated patients with MIBC treated with NAC with cisplatin, methotrexate, vinblastine (CMV) followed by RC (*n* = 60). cCR assessment included cystoscopy, urine cytology, and abdominal imaging (ultrasound, CT or MRI). cCR was observed in 10% (6/60) of evaluable patients, while pCR was achieved in 8.3% (5/60). In all, 50% (3/6) of patients with cCR had also pCR. Survival was significantly better for patients with partial or complete pathological response compared to non‐responders (*P* < 0.001), with 5‐year survival rates of 84% vs 44%, respectively.

### Meta‐Analysis: cCR and pCR Rates

**Fig. 1:**
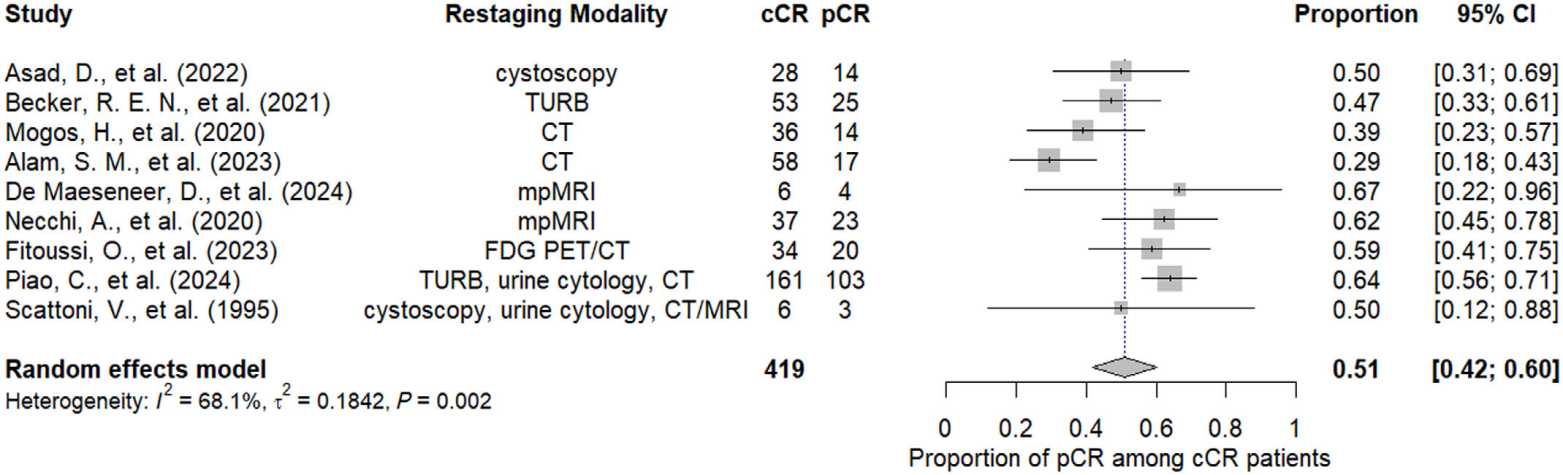
Forest plot of concordance between cCR and pCR.

Nine (*n* = 779) of the 13 eligible studies were included in the concordance analysis [19, 20, 21, 22, 24, 27, 28, 29, 31]; four studies were excluded due to incomplete reporting of cCR rates. The overall concordance between cCR and pCR was 0.51 (95% CI 0.42–0.60, Fig. 1). There was evidence of significant heterogeneity (*I* ^2^ = 68%); with leave‐one‐out analysis identifying one study as a major contributor to this heterogeneity [19] (File 3.1. in Data S1). After excluding this study, the pooled concordance increased marginally to 0.55 (95% CI 0.47–0.62) and heterogeneity was substantially reduced (*I* ^2^ = 37%) (Files 3.2. in Data S1). In an additional sensitivity analysis excluding a study in which perioperative immunotherapy rather than cisplatin‐based chemotherapy was administered [28], potentially contributing to heterogeneity, the concordance was 0.49 (95% CI 0.40–0.59, File 3.3. in Data S1), with substantial heterogeneity remaining among the other studies (*I* ^2^ = 71%).

**Fig. 2:**
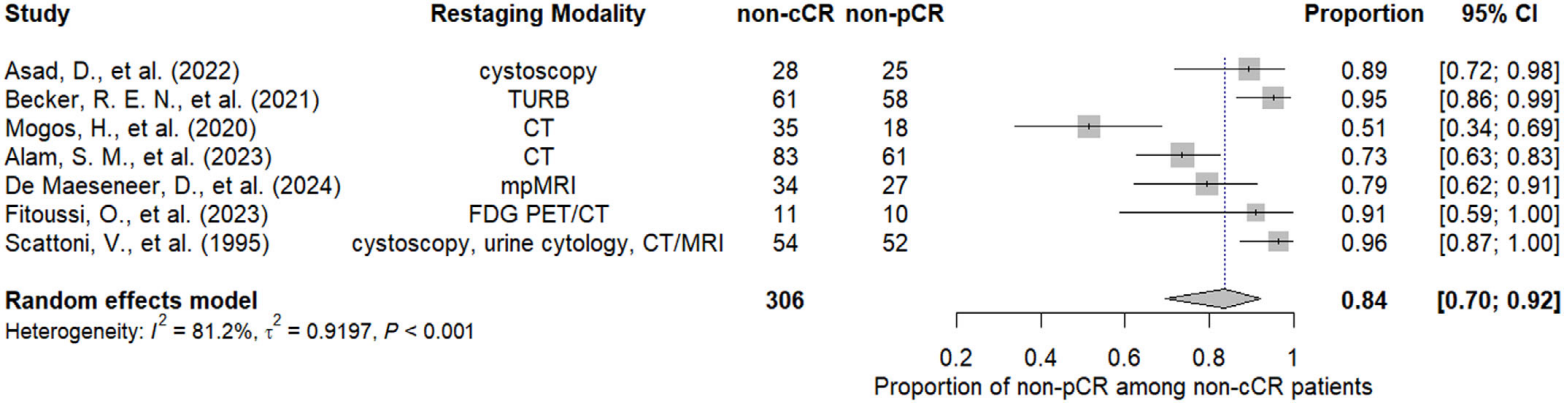
Forest plot of concordance between non‐cCR and non‐pCR.

Seven (*n* = 536) of the nine studies provided data for the concordance analysis between non‐cCR and non‐pCR: the overall concordance was 0.84 (95% CI 0.70–0.92, Fig. 2). There was evidence of significant heterogeneity (*I* ^2^ = 81%); with leave‐one‐out analysis identifying one study as a major contributor to this heterogeneity [27] (File 3.4. in Data S1). After excluding this study, the pooled concordance increased marginally to 0.87 (95% CI 0.77–0.93) with reduced heterogeneity (*I* ^2^ = 70%) (Files 3.5. in Data S1).

#### Subset‐Analysis: Concordance across Restaging Modalities

We conducted subset analyses stratified by restaging modality (non‐invasive modalities: CT, MRI, or FDG PET; invasive modalities: cystoscopy and TURB). In studies (*n* = 388, five studies) using non‐invasive restaging modalities (CT, mpMRI, FDG PET/CT), the pooled concordance between cCR and pCR was 0.48 (95% CI 0.34–0.63, File 4.1. in Data S1). In studies (*n* = 391, four studies) using restaging approaches that included invasive procedures (cystoscopy, TURB), the pooled concordance between cCR and pCR was 0.55 (95% CI 0.45–0.65, File 4.2. in Data S1).

#### Subset Analysis: cCR and Residual Disease

Four studies (*n* = 332) reported on patients who achieved cCR but had a residual pathological tumour classified as ≤pT1 (pTis, pTa, pT1) [20, 21, 27, 28]. In a subset analysis of these studies, the pooled proportion of patients with residual non‐muscle‐invasive disease was 0.27 (95% CI 0.20–0.36; File 5 in Data S1), indicating that approximately one‐quarter of patients with cCR still harboured non‐muscle‐invasive residual disease. Heterogeneity across these studies was low to moderate (*I* ^2^ = 38%).

## Discussion

Across the included studies with various restaging modalities to assess cCR, we observed remarkably high rates of false‐positive cCR. The pooled analysis revealed only moderate concordance between cCR and pCR, with pathological residual disease more than half of the patients were categorised to have had a cCR. This highlights the limitations of using cCR based on current staging methods with the current tools used in the studies as a surrogate biomarker for pCR in patients seeking bladder preservation therapies, supporting the need to identify validated more accurate criteria for cCR.

The heterogeneity in cCR assessment, ranging from imaging alone to invasive techniques, reflects the absence of a validated, reproducible and standardised protocol. However, none of the included studies indicated high sensitivity in detecting pCR using any of the tested cCR assessment methods. Cystoscopy and TURB failed to reliably predict pCR, with studies consistently reporting negative predictive values of 48% to 50% [20, 32]. Similarly, conventional imaging struggled to differentiate viable tumour from post‐NAC fibrosis, frequently missing nodal micro‐metastases [33]. While some modalities such as mpMRI showed numerically higher estimates, their sensitivity remained insufficient to consistently identify pCR [22], and other imaging modalities, such as CT, were also associated with particularly low sensitivity [19, 27]. Our analysis revealed that across both concordance analyses, CT consistently showed the lowest concordance, with substantial variation across studies, likely driven by methodological heterogeneity and inter‐observer variability in clinical response assessment. This highlights the need for more objective, valid, accurate, and reproducible biomarkers to guide treatment decisions.

Bladder‐sparing strategies for MIBC after cCR include trimodal therapy (TMT), PC, and radical TURB. TMT, integrating maximal TURB, radiotherapy with radiosensitisers, and often NAC, has demonstrated 5‐year metastasis‐free survival (MFS) and cancer‐specific survival rates of 74% and 85%, respectively, and a higher 5‐year OS compared with RC (77% vs 72%) [34]. PC, suitable for small solitary tumours, yields 5‐year OS of 57–79% but is non‐standard due to high recurrence risks [35, 36, 37]. Radical TUR, for TMT/RC‐ineligible patients, achieves ~76% 10‐year disease‐specific survival in select cT2 cases without CIS [38, 39]. Emerging NAC/immunotherapy approaches, like HCRN GU16‐257 (gemcitabine/cisplatin plus nivolumab), report 97% 2‐year MFS in cCR patients avoiding RC/TMT [13]. However, unreliable cCR assessment methods highlight the need for validated biomarker.

Emerging strategies, particularly urinary tumour DNA (utDNA), circulating tumour DNA (ctDNA), and artificial intelligence offer promising solutions to overcome current challenges in restaging patients with MIBC [40, 41, 42, 43]. Due to the direct interface between urine and urothelial tumours, utDNA represents a highly tumour‐specific and non‐invasive biomarker for detecting residual intravesical disease. Recent evidence suggests that utDNA outperforms traditional diagnostic tools, achieving high sensitivity and specificity in identifying high‐grade or invasive bladder cancer, even in early stages [44]. In contrast, ctDNA dynamics have demonstrated strong prognostic value for systemic disease, with clearance correlating with pathological downstaging and improved survival, while persistent ctDNA predicts residual disease and early recurrence [45, 46]. Notably, Christensen et al. [46] demonstrated that ultra‐deep sequencing of ctDNA achieved 100% sensitivity and 98% specificity in detecting metastatic relapse: all ctDNA‐positive patients had residual disease at RC, and all the patients with pCR were ctDNA‐negative, highlighting ctDNA's prognostic value. Although ctDNA remains valuable for monitoring systemic spread, utDNA may be more suitable for accurately identifying intravesical residual disease following NAC. Ongoing trials, such as the STARBURST project, are expected to further clarify the role of liquid biopsy and imaging strategies in restaging by randomising patients with cCR after NAC to either definitive local treatment or active surveillance, with intensive 3‐monthly assessments incorporating ctDNA and/or utDNA, cystoscopy and/or TUR, CT or PET‐CT, and MRI imaging [47].

That said, given the limitations of current restaging modalities and the potentially serious risks of undertreatment [48], RC with perioperative immunochemotherapy remains the standard of care for patients with non‐metastatic MIBC. The rise of novel neoadjuvant strategies, particularly immune checkpoint inhibitor‐based combinations, has shown promising potential in improving survival outcomes and pCR rates, which may ultimately expand opportunities for bladder preservation [3]. While these advances are encouraging, we found critical uncertainties regarding optimal cCR assessment. An optimal approach to cCR assessment may involve a combination of maximal TURB, mpMRI, and molecular biomarkers to enhance diagnostic accuracy and support treatment stratification. Until definitive evidence from ongoing bladder preservation trials establishes both the safety thresholds and optimal surveillance protocols, RC after NAC remains the standard curative approach for MIBC outside clinical trials [13, 48, 49, 50]. From a clinical perspective, the use of cCR as a surrogate endpoint for pCR carries substantial implications for patient safety, as it implies the absence of residual tumour and may influence decisions to omit RC, which remains associated with significant survival benefits as the current standard of care [1]. Therefore, an ideal surrogate endpoint would require (near‐)perfect agreement with pCR, although such accuracy is rarely achievable in clinical practice, especially considering that even pathological assessment itself is not without limitations. However, in our pooled analysis the concordance between cCR and pCR was only moderate (approximately 0.51–0.55), underscoring that current clinical assessment methods are insufficiently reliable to replace pathological confirmation and should be interpreted with caution in the context of BSP strategies.

The findings of this meta‐analysis should be interpreted considering certain limitations. First, the included studies were highly heterogeneous in design, with most being retrospective and featuring small patient cohorts, introducing potential selection bias and limits the generalisability of the results. Second, the timing between restaging and RC was inconsistently reported, with some studies performing assessments immediately before surgery and others after NAC completion but before the final cycle, introducing variability in response evaluation. The number of chemotherapy cycles administered varied across studies, predominantly cisplatin‐based; however, information on the distribution of cycles among individual patients was frequently not reported or stratified, precluding an analysis of their potential impact on cCR or concordance with pCR. Third, while the definition of cCR was restricted to cT0, the restaging modality varied significantly across studies, ranging from imaging alone (CT, MRI, or PET/CT) to invasive techniques (cystoscopy, TURB) or combined approaches. Heterogeneity in neoadjuvant therapy regimens across studies may have influenced pCR rates and, consequently, the observed concordance between clinical and pathological assessments. Substantial heterogeneity initially precluded meaningful subgroup analyses by restaging method, so we performed a simplified comparison between invasive and non‐invasive procedures, which did not reveal notable differences in concordance.

## Conclusion

Our analysis demonstrates that existing methods show poor and highly variable concordance with pathological outcomes, highlighting that we currently lack reliable tools to assess cCR with sufficient accuracy. This diagnostic uncertainty makes cCR an inadequate standalone basis for treatment decisions. While ongoing trials, particularly those investigating utDNA and ctDNA, hold promise for improving cCR evaluation, RC remains the standard of care outside clinical trials. Emerging data on perioperative immunotherapy and ongoing trials evaluating enfortumab vedotin plus pembrolizumab, are expected to further shape the neoadjuvant landscape and influence future pCR benchmarks. To safely advance BSP strategies, future research must urgently focus on developing and validating better, easy, fast, and cheap biomarkers of cCR at the bladder and systemic level.

## Disclosure of Interests

Shahrokh F. Shariat received the following: Honoraria: Astellas, AstraZeneca, BMS, Ferring, Ipsen, Janssen, MSD, Olympus, Pfizer, Roche, Takeda. Consulting or Advisory Role: Astellas, AstraZeneca, BMS, Ferring, Ipsen, Janssen, MSD, Olympus, Pfizer, Pierre Fabre, Roche, Takeda. Speakers Bureau: Astellas, Astra Zeneca, Bayer, BMS, Ferring, Ipsen, Janssen, MSD, Olympus, Pfizer, Richard Wolf, Roche, Takeda. Margit Fisch received the following: Honoraria: Astellas, Apogepha. Grant: Boston Scientific. Bernadett Szabados received the following: Research and travel funding: Roche, Genentech, MSD, Pfizer, BMS, Photocure. Honoraria: Merck, Roche, Pfizer, Ellipses, Ipsen, Janssen, Photocure, Astellas, AstraZeneca. Marcin Miszczyk was supported by the European Urological Scholarship Programme (EUSP) Scholarship of the European Association of Urology (EAU). The other authors declare no conflicts of interest associated with this manuscript.

## Funding

The authors have nothing to report.

## Author Contributions

Navid Roessler, Alessandro Dematteis, Marcin Miszczyk, Keiichiro Miyajima, Shota Inoue, and Bernadett Szabados contributed to protocol/project development, data collection, data analysis, and manuscript writing/editing. Navid Roessler, Alessandro Dematteis, Paolo Gontero, Keiichiro Miyajima, Shota Inoue, Katharina Oberneder, Markus von Deimling, Malte W. Vetterlein, Victor M. Schuettfort, Pawel Rajwa, Bernadett Szabados, Pierre I. Karakiewicz, Jeremy Yuen‐Chun Teoh, Margit Fisch, and Marcin Miszczyk contributed to manuscript writing/editing. Marcin Miszczyk, and Shahrokh F. Shariat contributed to supervision and manuscript editing.

## Supporting information


Data S1.
File 1 Preferred Reporting Items for Systematic Reviews and Meta‐analyses (PRISMA) flow diagram for new systematic reviews, which included searches of databases and registers only.
**File 2** Detailed search strategy for the databases.
**File 3** Meta‐analysis of clinical and pathological complete response rates.
**File 4** Concordance between cCR and pCR (invasive vs non‐invasive restaging modalities).
**File 5** Subset analysis: clinical complete response and residual disease (forest/funnel).
**File 7** The PICO(S) framework.
**File 8** The MeaSurement Tool to Assess systematic Reviews 2 (AMSTAR 2) checklist.
