# Supplementary material for: Clinical complete response as a surrogate for pathological response in bladder cancer: a systematic review and meta‐analysis
Source: BJU Int. 2026 Mar 16;138(2):186–95. doi: 10.1111/bju.70214 (PMC13371391; doi:10.1111/bju.70214)

Table of Contents

[**1.** **Supplementary File 1: Preferred Reporting Items for Systematic Reviews and Meta-analyses (PRISMA) - flow diagram for new systematic reviews which included searches of databases and registers only** 2](#_Toc213573188)

[**2.** **Supplementary File 2: Detailed Search Strategy for the Databases** 3](#_Toc213573189)

[**3.** **Supplementary File 3: Meta-analysis of Clinical and Pathological Complete Response Rates** 6](#_Toc213573190)

[**4.** **Supplementary File 4: Concordance between cCR and pCR (Invasive vs. Non-Invasive Restaging Modalities)** 11](#_Toc213573191)

[**5.** **Supplementary File 5 - Subset Analysis: Clinical Complete Response and Residual Disease (Forest/Funnel)** 13](#_Toc213573192)

[**6.** **Supplementary File 6: Risk of Bias according to ROBINS-I tool** 14](#_Toc213573193)

[**7.** **Supplementary File 7: PICO(S) Framework** 15](#_Toc213573194)

[**8.** **Supplementary File 8: AMSTAR 2 Checklist** 16](#_Toc213573195)

# **Supplementary File 1: Preferred Reporting Items for Systematic Reviews and Meta-analyses (PRISMA) - flow diagram for new systematic reviews which included searches of databases and registers only**

**Report included (n=13)**

**Studies included (n=13)**

**Identification of studies via databases and registers**

**Screening**

Records screened

(n=1947)

Records excluded

(n=1774)

Reports sought for retrieval

(n=173)

Reports not retrieved

(n=0)

Reports assessed for eligibility

(n=173)

Reports excluded

- RT as definitive therapy (n=1)
- no difference between UTUC and BCa

(n=1)

- partial cystectomy

(n=1)

- no cCR

(n=132)

- no pCR

(n=25)

**Included**

**Identification**

Records identified by database searching (n=2798):

MEDLINE: (n=851)

Embase: (n=980)

Web of Science (n=967)

Records removed *before screening*:

- Review (n=289)
- Case-Report

(n=53)

- Conf. Abstract/Editorial

(n=53)

- non-English (n=83)
- Duplicate removal (n=373)

Source: Page MJ, et al. BMJ 2021;372:n71. doi: 10.1136/bmj.n71.

This work is licensed under CC BY 4.0. To view a copy of this license, visit <https://creativecommons.org/licenses/by/4.0/>

# **Supplementary File 2: Detailed Search Strategy for the Databases**

**MEDLINE (PubMed) – 25/06/03**

| 1 | (  "bladder cancer*"[Title/Abstract]  OR "urothelial carcinoma*"[Title/Abstract]  OR "urothelial cancer*"[Title/Abstract]  OR "bladder carcinoma*"[Title/Abstract]  OR "Urinary Bladder Neoplasms"[MeSH Terms]  OR "Carcinoma, Transitional Cell"[MeSH Terms]  ) | 93878 |
| --- | --- | --- |
| 2 | (  "neoadjuvant"[Title/Abstract]  OR "preoperative"[Title/Abstract]  OR "perioperative"[Title/Abstract]  OR "Neoadjuvant Therapy"[MeSH Terms]  OR "NAC"[Title/Abstract]  OR ("neoadjuvant"[Title/Abstract] AND "immunotherapy"[Title/Abstract])  ) | 578820 |
| 3 | (  "restaging"[Title/Abstract]  OR "tumor response"[Title/Abstract]  OR "response assessment"[Title/Abstract]  OR "treatment response"[Title/Abstract]  OR "clinical response"[Title/Abstract]  OR "clinical complete response"[Title/Abstract]  OR "cCR"[Title/Abstract]  OR "pathologic response"[Title/Abstract]  OR "pathological response"[Title/Abstract]  OR "pathologic complete response"[Title/Abstract]  OR "pathological complete response"[Title/Abstract]  OR "pCR"[Title/Abstract]  OR "complete response"[Title/Abstract]  OR "cT0"[Title/Abstract]  OR "ypT0"[Title/Abstract]  ) | 818835 |
|  | 1 AND 2 AND 3 | 851 |

**Embase – 25/06/03**

| 1 | (  'urinary bladder neoplasm'/exp  OR 'bladder cancer'/exp  OR 'urothelial carcinoma'/exp  OR 'transitional cell carcinoma'/exp  ) | 148547 |
| --- | --- | --- |
| 2 | (  'neoadjuvant therapy'/exp  OR 'preoperative care'/exp  OR 'perioperative period'/exp  OR 'neoadjuvant immunotherapy'/exp  ) | 1393759 |
| 3 | (  'restaging'/exp  OR 'tumor response'/exp  OR 'treatment response'/exp  OR 'response evaluation'/exp  OR 'clinical response'/exp  OR 'complete clinical response'/exp  OR 'pathological response'/exp  OR 'complete pathological response'/exp  OR 'complete remission'/exp  ) | 429030 |
|  | 1 AND 2 AND 3 | 980 |

**Web of Science – 25/06/03**

| 1 | TS=(  "bladder cancer*"  OR "urothelial carcinoma*"  OR "urothelial cancer*"  OR "bladder carcinoma*"  OR "urinary bladder neoplasm*"  OR "transitional cell carcinoma*"  )  AND  TS=(  "neoadjuvant"  OR "preoperative"  OR "perioperative"  OR "neoadjuvant therapy"  OR "NAC"  OR ("neoadjuvant" AND "immunotherapy")  )  AND  TS=(  "restaging"  OR "tumor response"  OR "response assessment"  OR "treatment response"  OR "clinical response"  OR "clinical complete response"  OR "cCR"  OR "pathologic response"  OR "pathological response"  OR "pathologic complete response"  OR "pathological complete response"  OR "pCR"  OR "complete response"  OR "cT0"  OR "ypT0"  ) | 967 |
| --- | --- | --- |

# **Supplementary File 3: Meta-analysis of Clinical and Pathological Complete Response Rates**

- 1.
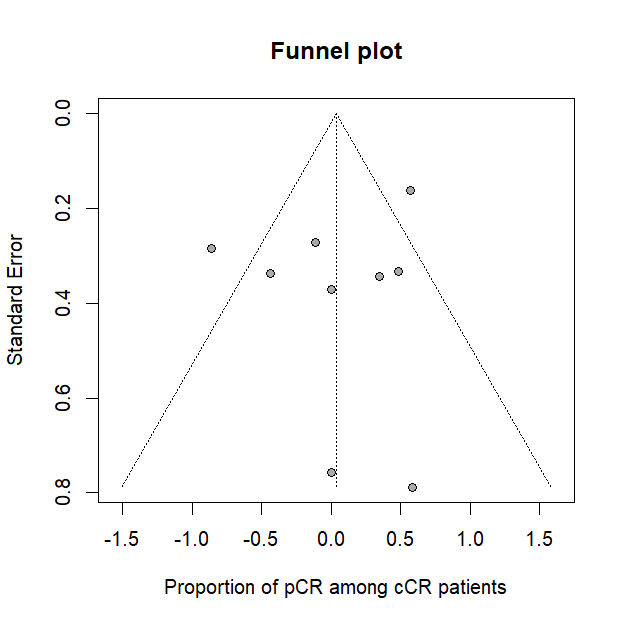
**Concordance between cCR and pCR (Funnel/Leave-One-Out)**


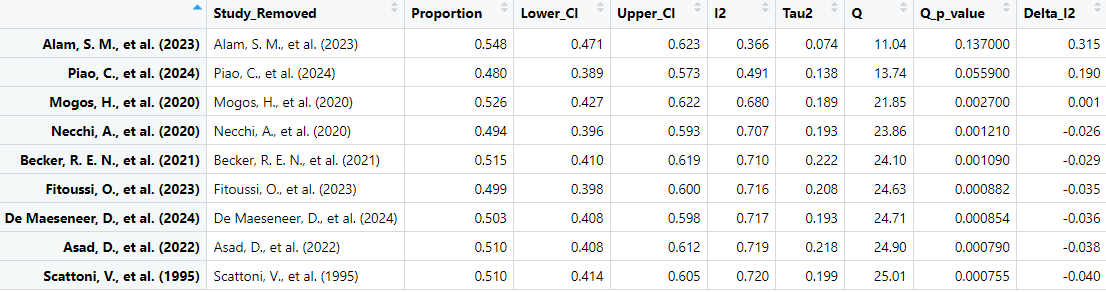


- 1. **Concordance between cCR and pCR after sensitivity-based study exclusion (Forest/Funnel)**

**
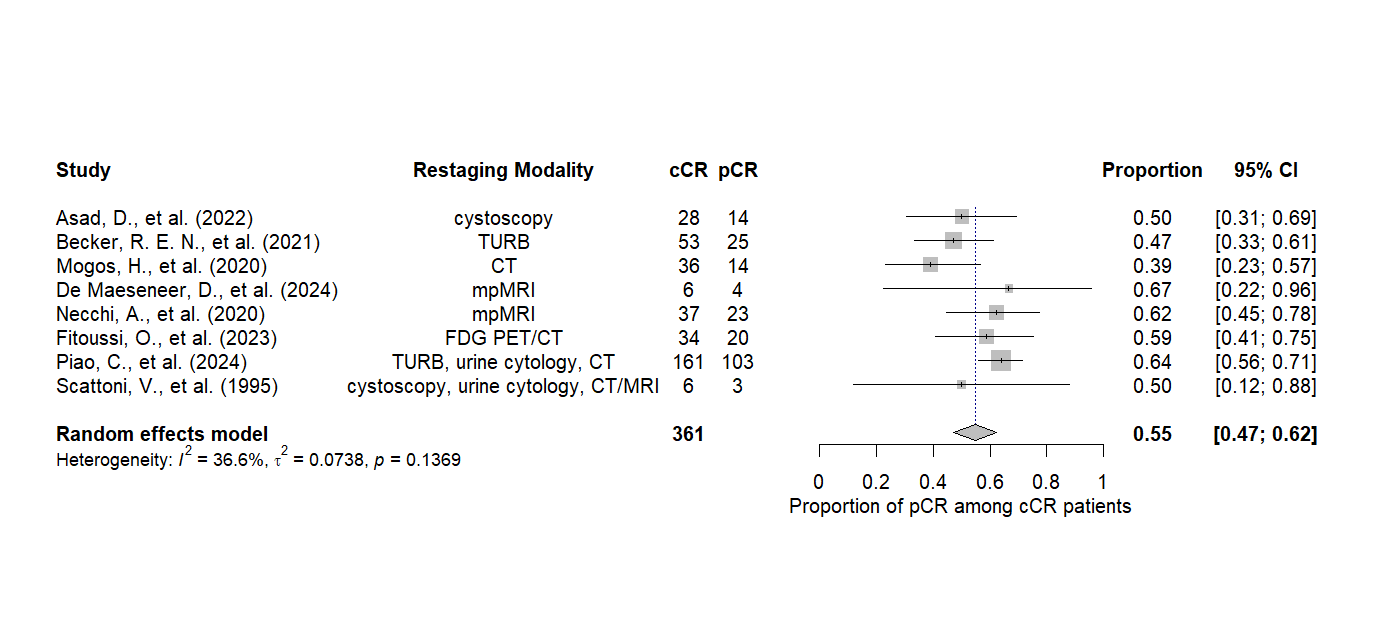

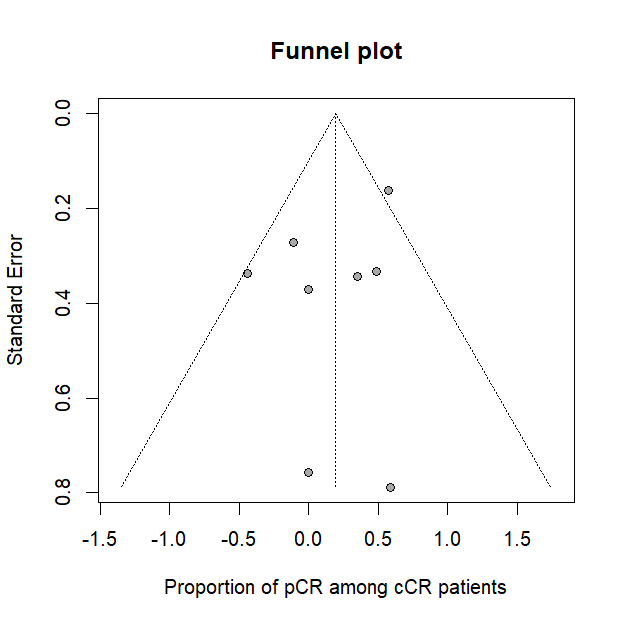
**

- 1.
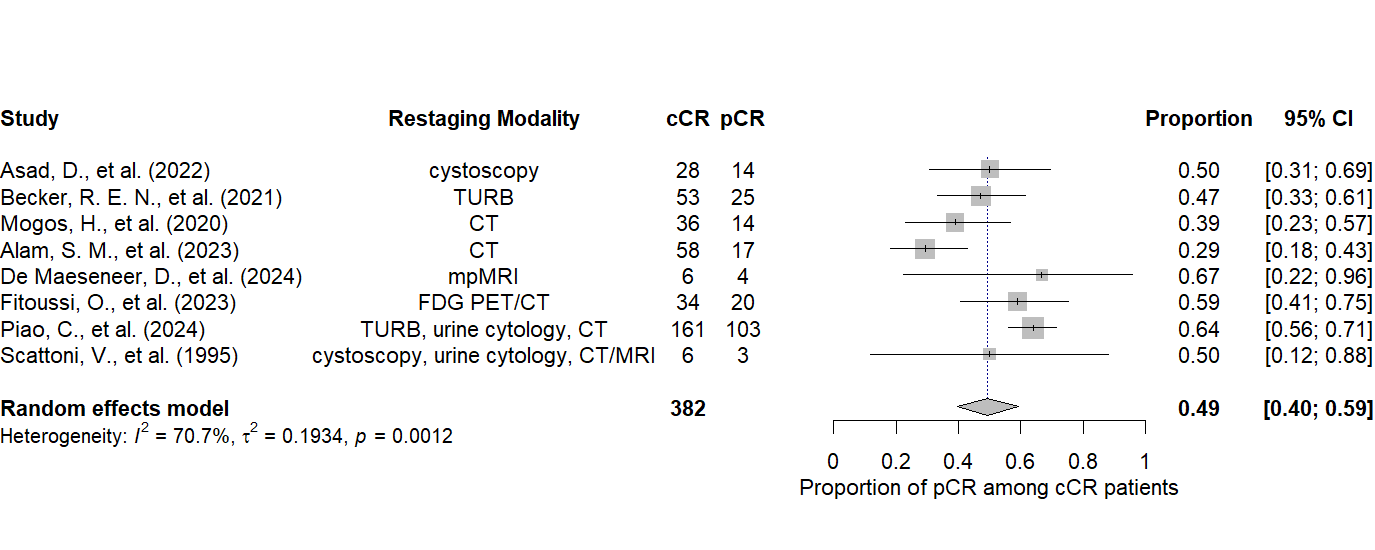
**Concordance between cCR and pCR after excluding a study with perioperative immunotherapy as a potential source of heterogeneity (Forest/Funnel)**

**
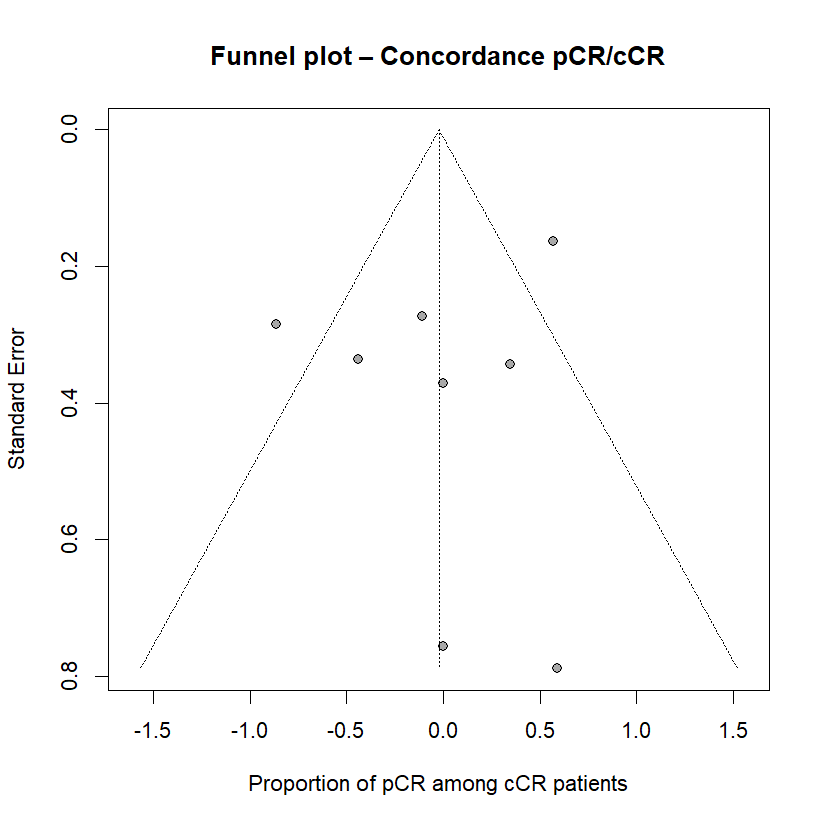
**

- 1. **Concordance between non-cCR and non-pCR**
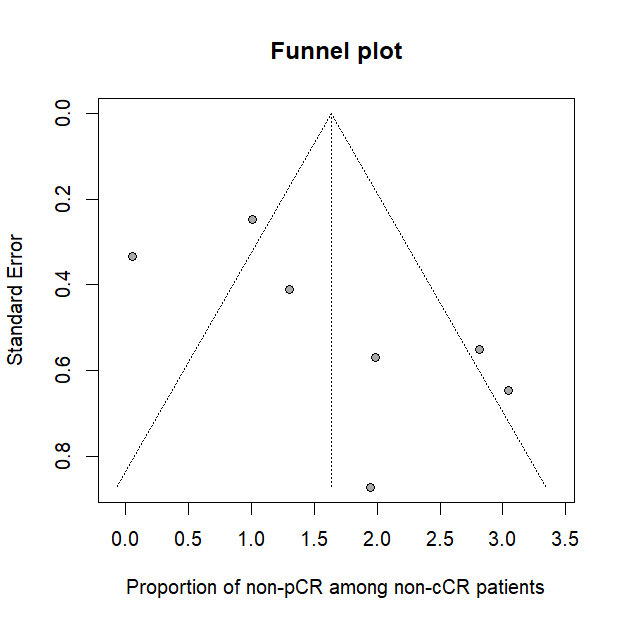
**(Funnel/Leave-One-out)**

**
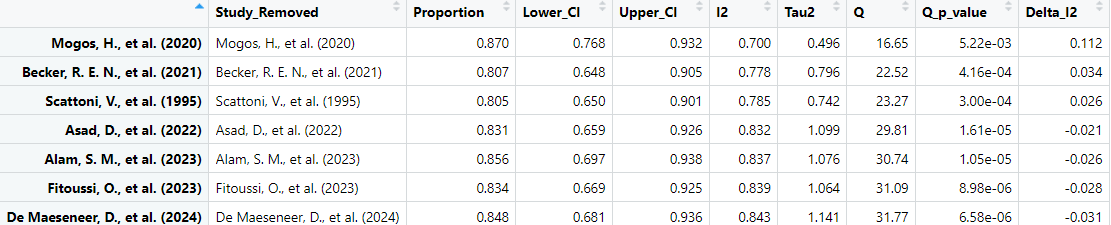
**

- 1. **Concordance between non-cCR and non-pCR after sensitivity-based study exclusion (Forest/Funnel)**

**
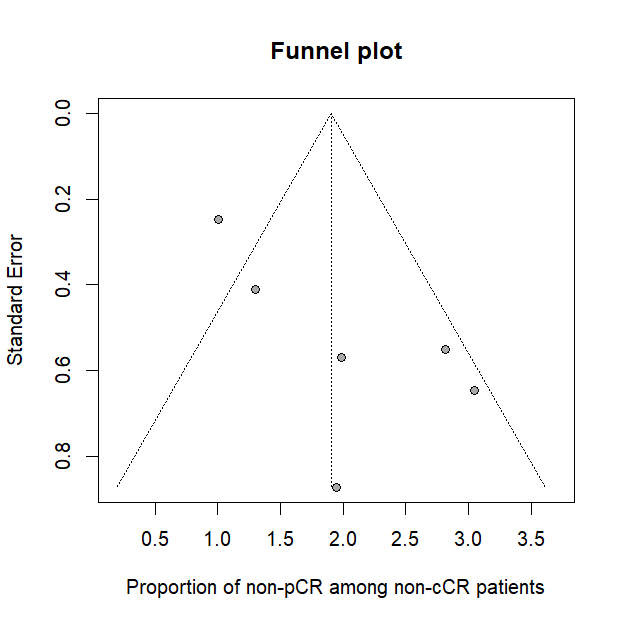

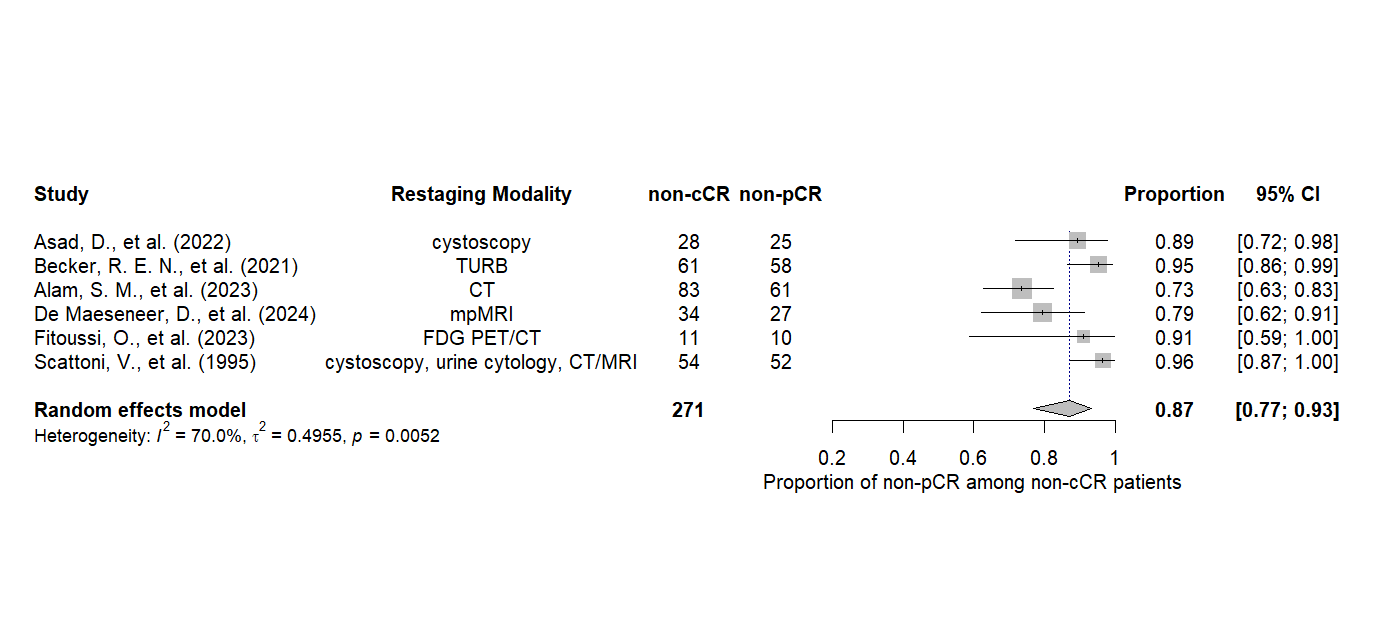
**

# **Supplementary File 4: Concordance between cCR and pCR (Invasive vs. Non-Invasive Restaging Modalities)**

- 1. **Non-Invasive Restaging Modalities (Forest/Funnel)**

**
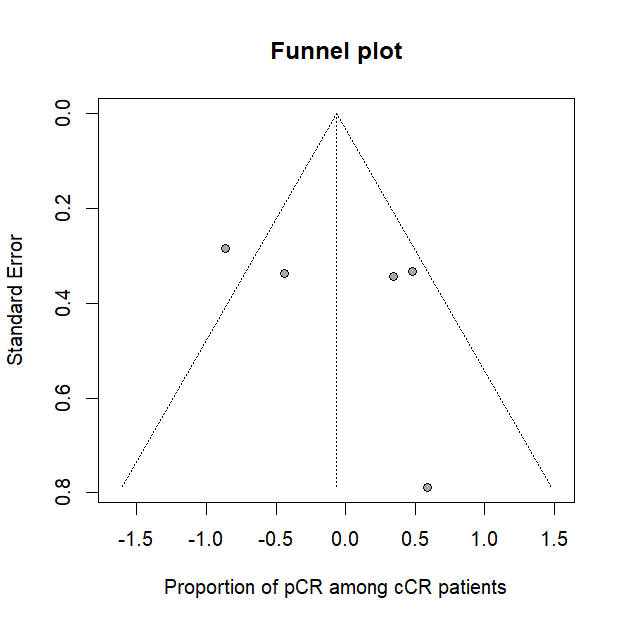

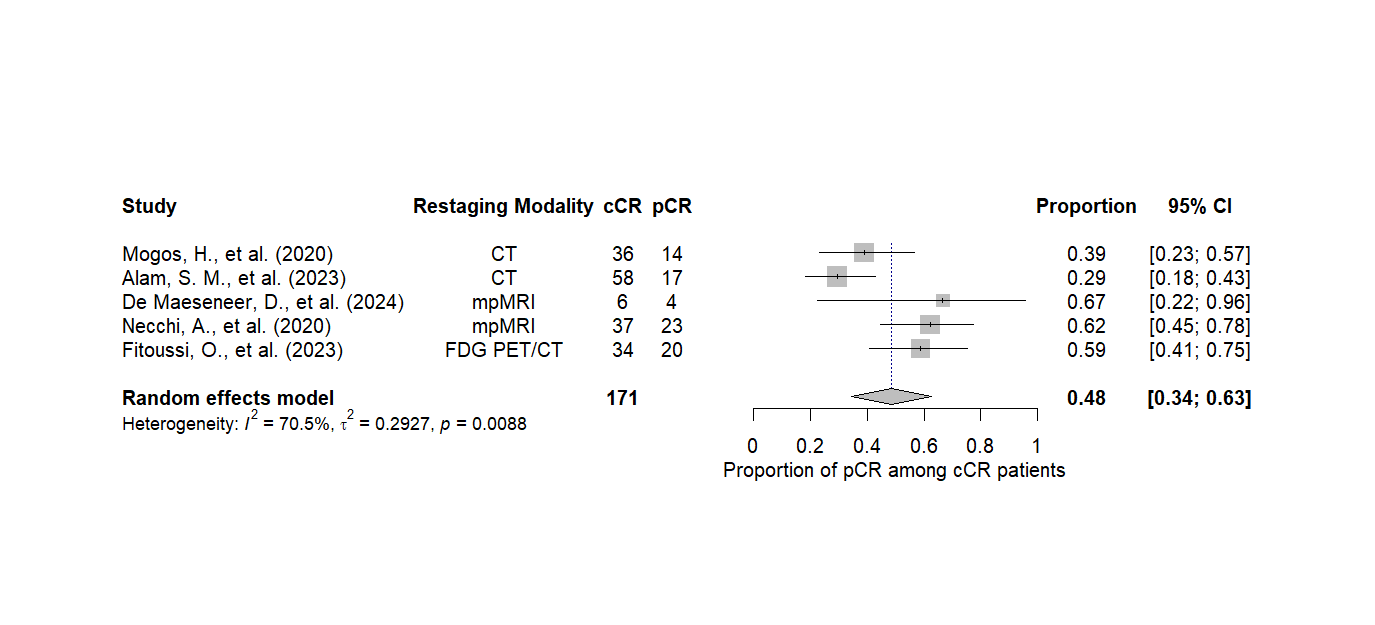
**

- 1. **Invasive Restaging Modalities (Forest/Funnel)**

**
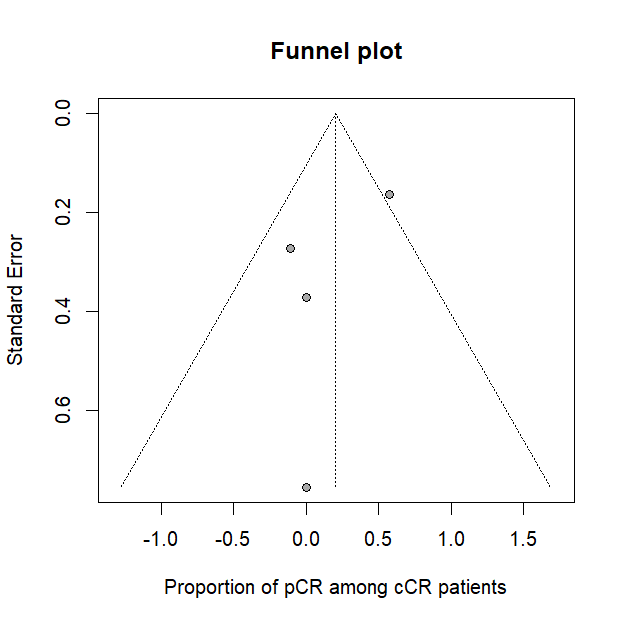
**

**
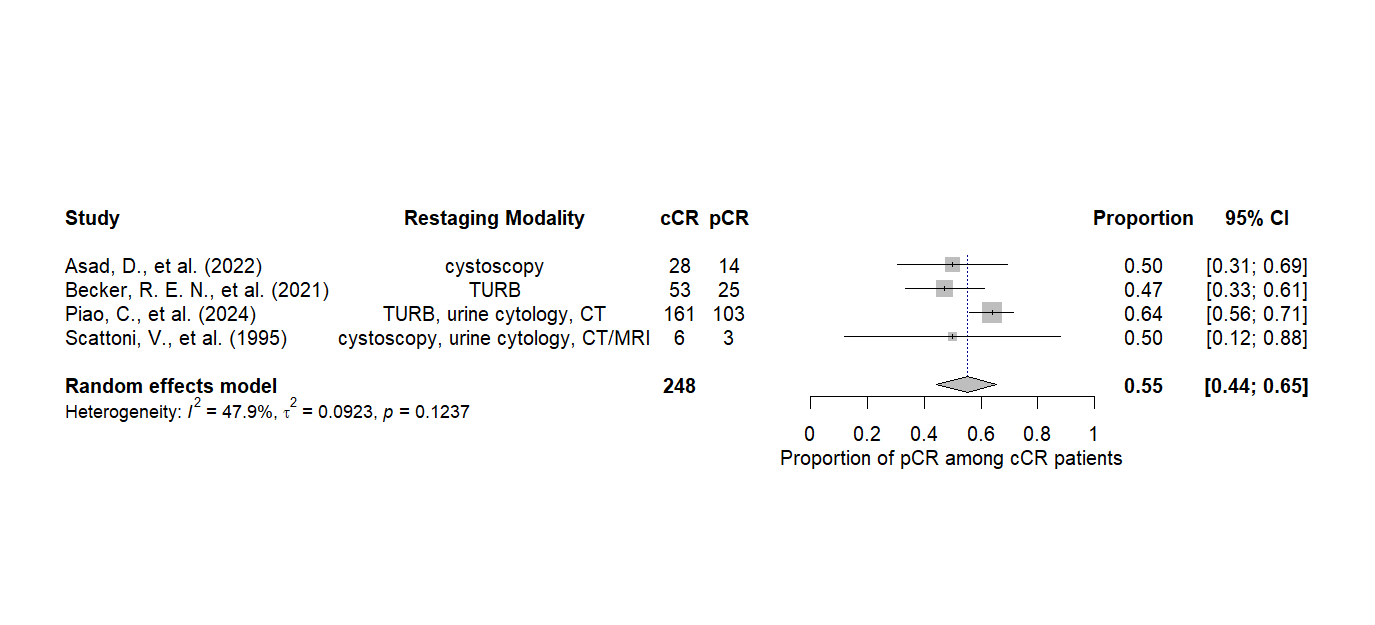
**

# **Supplementary File 5 - Subset Analysis: Clinical Complete Response and Residual Disease (Forest/Funnel)**

**
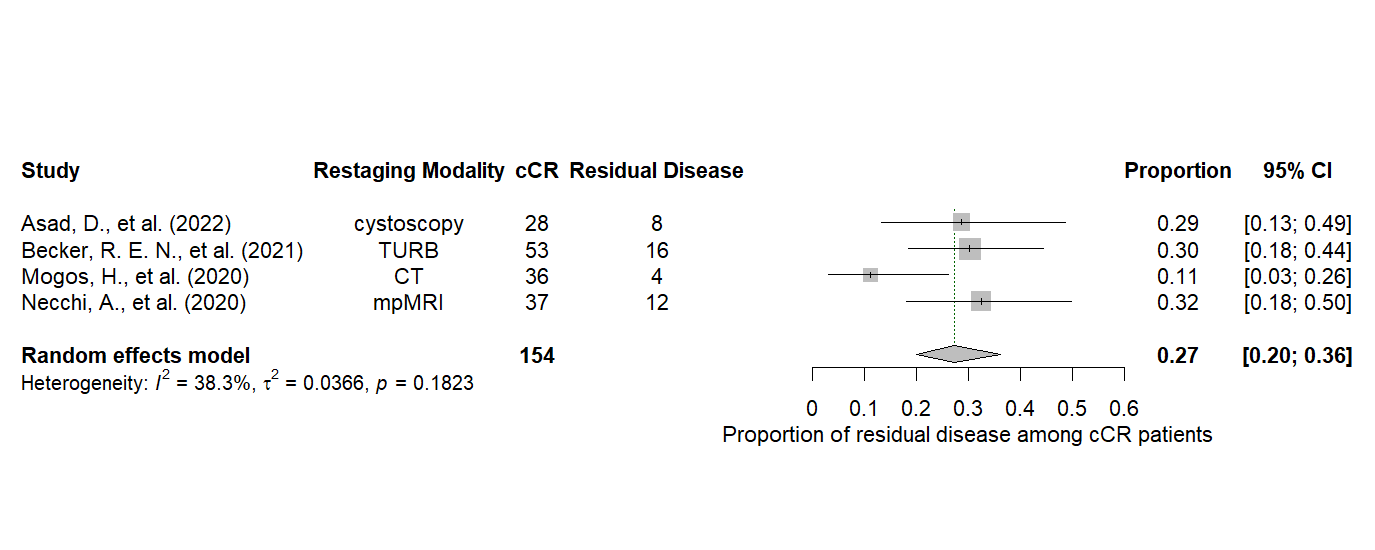
**

**
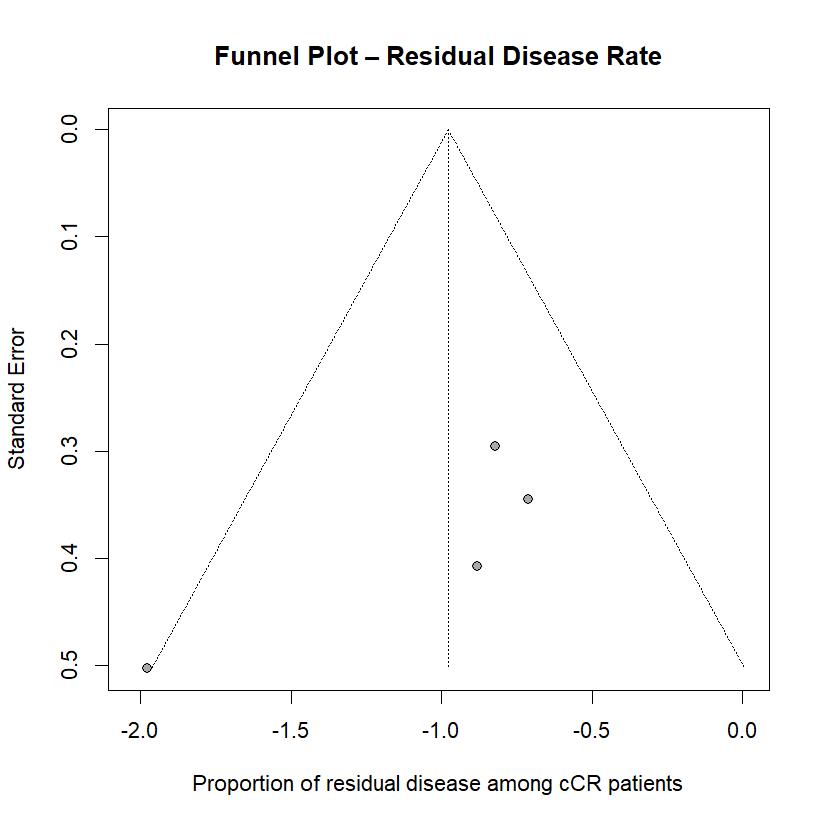
**

# **Supplementary File 6: Risk of Bias according to ROBINS-I tool**


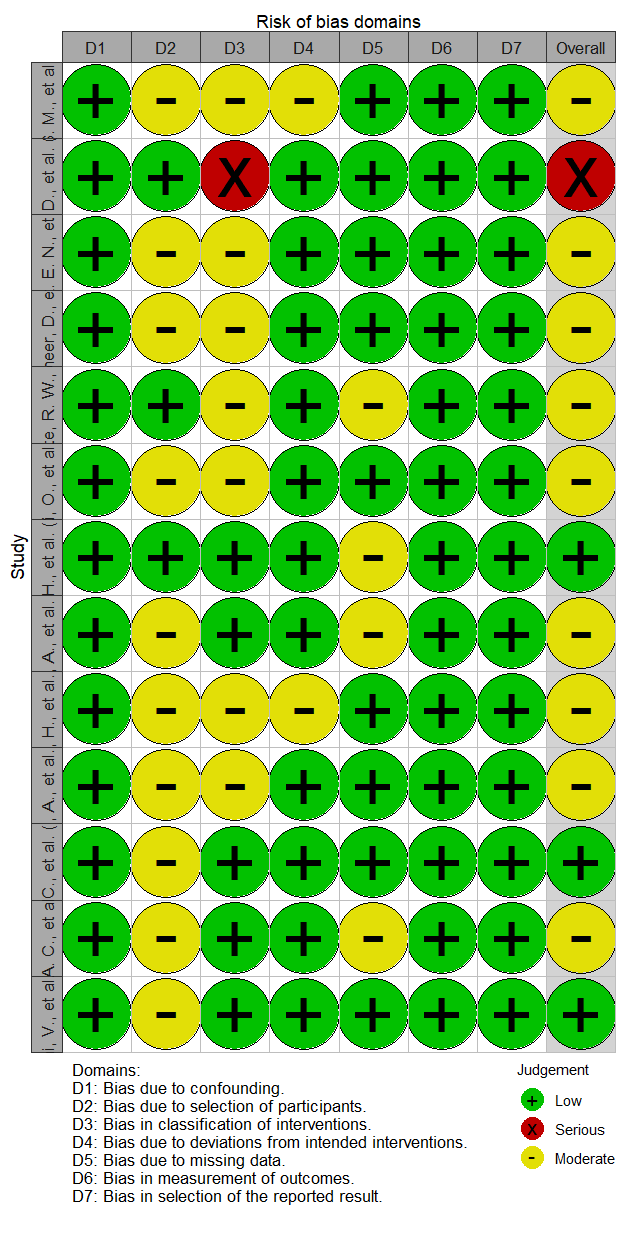


# **Supplementary File 7: PICO(S) Framework**

P:

Adults (≥18 years) diagnosed with muscle-invasive bladder cancer (MIBC), clinical stages cT2–T4a, N0, M0.

Patients who have received neoadjuvant chemotherapy or immunotherapy prior to surgery.

I:

Assessment of clinical complete response (cCR, pT0) through cystoscopy, imaging modalities (e.g., CT, MRI), and/or urine cytology following neoadjuvant therapy.

C:

Pathological assessment of response (pCR, ypT0N0) based on surgical specimen analysis after cystectomy.

O:

Concordance between clinical complete response (cCR) and pathological complete response (pCR), including sensitivity, specificity, positive predictive value (PPV), and negative predictive value (NPV).

S:

Prospective and retrospective reports, randomized-controlled trials, post-hoc and subsequent analyses of trials

# **Supplementary File 8: AMSTAR 2 Checklist**


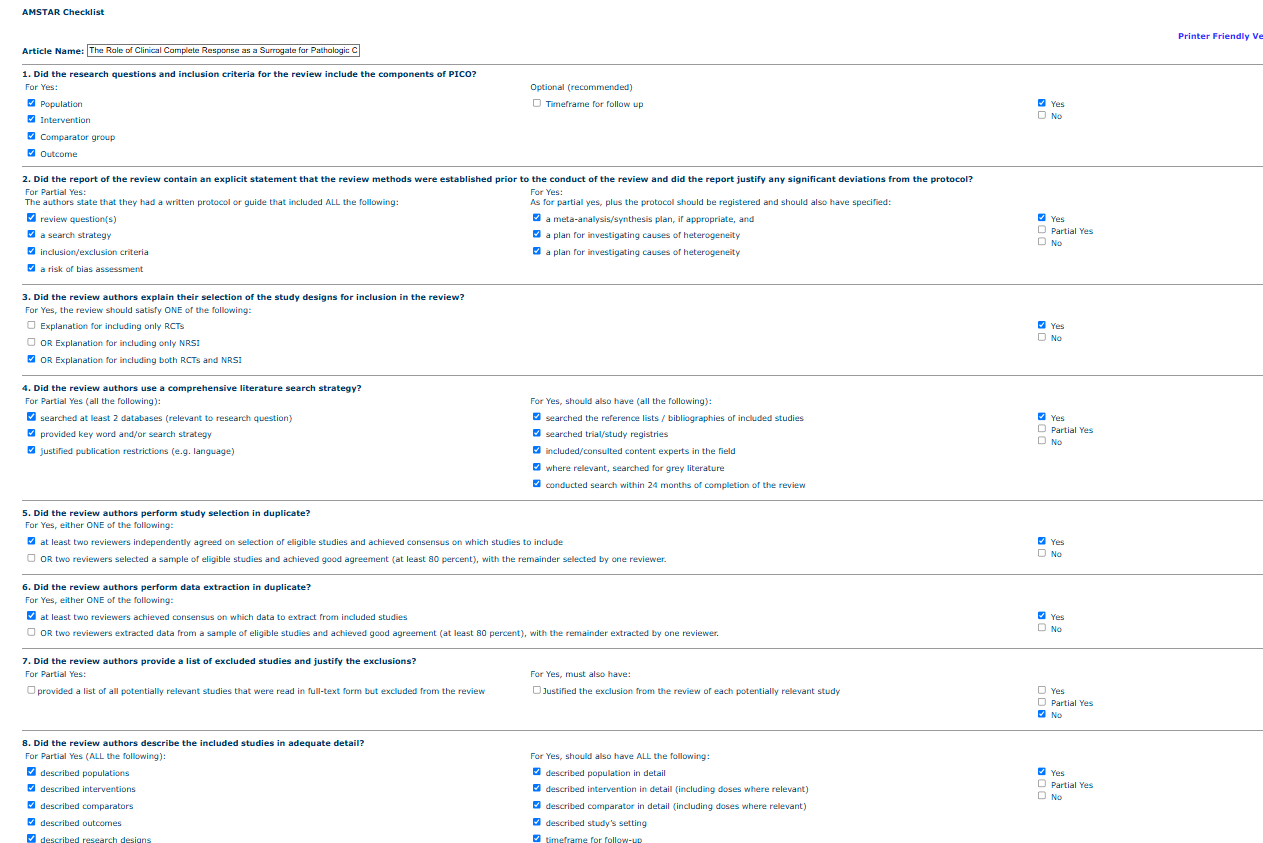

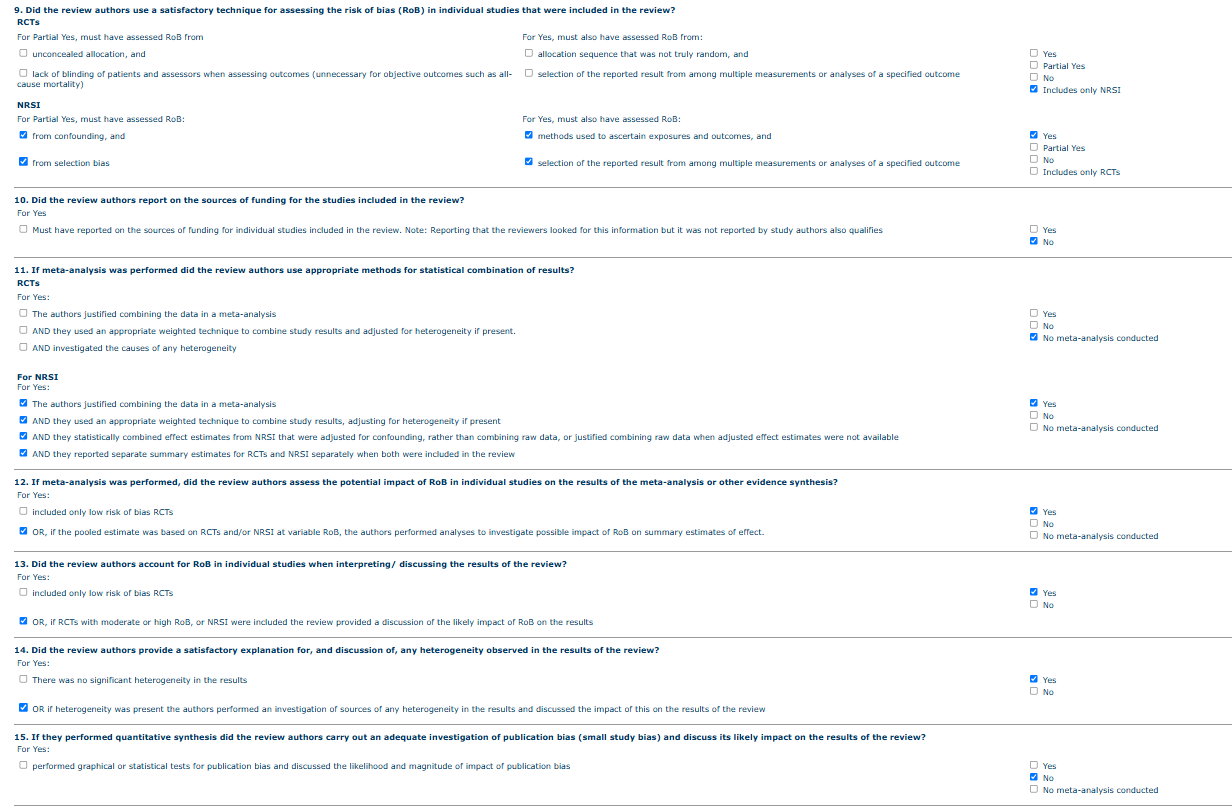

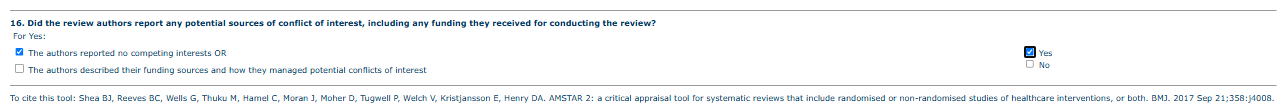

Supplement: Supplementary file 1 — Data S1. File 1 Preferred Reporting Items for Systematic Reviews and Meta‐analyses (PRISMA) flow diagram for new systematic reviews, which included searches of databases and registers only. File 2 Detailed search strategy for the databases. File 3 Meta‐analysis of clinical and pathological complete response rates. File 4 Concordance between cCR and pCR (invasive vs non‐invasive restaging modalities). File 5 Subset analysis: clinical complete response and residual disease (forest/funnel). File 7 The PICO(S) framework. File 8 The MeaSurement Tool to Assess systematic Reviews 2 (AMSTAR 2) checklist. [file BJU-138-186-s001.docx]
